# Supplementary material for: Reliability, Validity and Responsiveness of the Polish Version of the Western Ontario Shoulder Instability Index (WOSI-PL) in Patients after Arthroscopic Repair for Shoulder Instability
Source: Int J Environ Res Public Health. 2022 Oct 27;19(21):14015. doi: 10.3390/ijerph192114015 (PMC9659089; doi:10.3390/ijerph192114015)
Supplement: Supplementary file 1 [file ijerph-19-14015-s001.zip › ijerph-1922332-supplementary.pdf]

Table S1. Measurement properties of the WOSI from the original article and subsequent validation studies.

Study	N	Internal consistency	ICC (interval)	Construct validity	SEM & SDC/MDC	MCID	SRM & ES
Kirkley et al.<br>1998 [3]<br>(original WOSI)	Total: N=300<br>Not specified	N = 33<br>item reduction	N=51<br>0.95 (2 weeks)<br>Domains:<br>0.72- 0.94<br>N = 18<br>0.91 (3 months)<br>Domains: 0.78- 0.91	N=47<br>DASH r = 0.77<br>UCLA r = 0.65<br>Constant r = 0.59<br>Rowe r = 0.61<br>ASES r = 0.55<br>SF12 PC r = 0.66<br>SF-12 MC r = 0.12<br>ROM r = 0.39	NA	NA	SRM 0.93
Salomonsson et al.<br>2009 [9]<br>(The Swedish version)	Total: N=99<br>22 surgery<br>32 closed reduction/surgery<br>45 healthy	N = 22<br>Preoperatively:<br>$\alpha = 0.89$<br>Domains: 0.56-0.89<br>Postoperatively:<br>$\alpha = 0.90$<br>Domains: 0.79-0.95	N = 32<br>0.94 (2 months)<br>Domains: 0.85-0.91	N = 22<br>VAS function r = 0.80 EQ-5D r = 0.44<br>Rowe r = 0.59	NA	NA	N = 22<br>SRM = 1.40<br>ES = 1.67
Hofstaetter et al.<br>2009 [10]<br>(The German version)	Total: N=86<br>24 surgery<br>25 closed reduction/ surgery<br>37 healthy	N = 24<br>$\alpha = 0.92$<br>Domains: 0.88-0.90	N = 25<br>0.92 (24–72 hours)<br>Domains: 0.87-0.93	N= 49<br>Rowe r = 0.627<br>UCLA r = 0.609<br>Constanst r = 0.590<br>SF-36<br>PF r = 0.44<br>SF r = 0.32<br>GH r = 0.34<br>MH r = 0.38<br>V r = 0.33<br>RE r = 0.32<br>RF r = 0.39<br>BP r = 0.56	NA	NA	NA
**Study**	**N**	**Internal consistency**	**ICC (interval)**	**Construct Validity**	**SEM & SDC/MDC**	**MCID**	**SRM & ES**

Drerup et al.<br>2010 [11]<br>(The German version)	Total: N=29<br>SI - not specified	N = 29<br>α = 0.89<br>Domains: 0,59-0.83	N = 29<br>0.87 (10 days)<br>Domains: 0.73-0,90	N=29<br>ASES r = 0,58	NA	NA	NA
Hatta et al.<br>2011 [12]<br>(The Japanese version)	Total: N = 85<br>SI:<br>76 traumatic SI<br>9 atraumatic SI	N = 85<br>α = 0.84<br>Domains: NA	N = 59<br>0.91 (2 weeks)<br>Domains: 0.64-0.86	N = 85<br>Quick DASH r = 0.63<br>Rowe Score r = 0.42<br>SF-36<br>PF r = 0.36<br>SF r = 0.10<br>GH r = 0.27<br>MH r = 0.26<br>V r = 0.28<br>RE r = 0.16<br>RF r = 0.26<br>BP r = 0.34	NA	NA	NA
Cacchio et al.<br>2011 [13]<br>(The Italian version)	Total: N=64<br>SI caused by a first-time traumatic anterior dislocation, treated by a rehabilitation protocol	N = 64<br>α = 0.93<br>Domains: NA	N = 59<br>0.95 (3 days)<br>N = 20<br>0.92 (14 weeks)<br><br>Domains: NA	N = 64<br>DASH r = 0.79<br>SF-36 r = 0.11<br>SF-36 PC r = 0.33<br>SF-36 MC r = 0.05	N = 64<br>SEM = 71 (3.4%)<br>SDC = 196 (9.3%)<br>Domains: NA	N = 59<br>MCID 400	N = 39<br>SRM = 1.94<br>ES = 1.47
Wiertsema et al.<br>2014 [15]<br>(The Dutch version)	Total: N = 52<br>22 traumatic SI<br>10 non-traumatic SI 10 post surgery	N = 52<br>α = 0.95<br>Domains: 0.88 - 0.95	N = 52<br>0.91 (25 days ± 17.3; 10–100 days)<br>Domains: 0.79-0.90	NA	SEM = 130.6 (6.2%)<br>Domains: 33.8-46.2<br>SDC = 362.0 (17.3 %)<br>Domains: 93.7-128.0	NA	NA

Study	N	Internal consistency	ICC (interval)	Construct validity	SEM & SDC/MDC	MCID	SRM & ES
Linde et al. 2014 [16] (The Dutch version)	Total: N = 138 anterior dislocations	N = 138 α = 0.96 Domains: 0.93-0.95	N = 99 0.92 (13 days; 5–30 days) Domains: 0.88-0.90	N = 138 OSIS r = 0.82 SST r = −0.66 OSS r = 0.79 DASH r = 0.81 SF-36: PF r = −0.69 RF r = −0.60 RE r = −0.48 MH r = −0.28 V r = −0.39 SF r = −0.51 BP r = −0.76 GH r = −0.36	N = 99 SEM = 8.3% Domains: 8.3- 10.1% SDC = 23.0% Domains: 23.1 -28.1%	NA	NA
Gaudelli et al. 2014 [17] European and North American French-speaking populations	Total: N = 144 awaiting shoulder stabilization surgery	NA	N = 144 0.84 (16 days; range: 3–357 days) N = 116 0.87 (range: 6 to 14 days) Domains: NA	N = 144 QuickDASH r = 0.65 Walch-Duplay r = −0.31	NA	NA	N = 49 SRM 1.55
Yuguero et al. 2016 [18] (The Spanish version)	Total: N = 79 N = 21 traumatic shoulder dislocation and treated by closed reduction N = 37 SI N= 21 healthy	N = 79 α = 0.96 Domains: 0.86-0.94	N = 42 0.95 Domains: 0.97-0.98	ROWE r = 0.745 EQ-5D-5L r = 0.471	N = 42 SEM = 23% Domains: NA MDC = 76% Domains: NA		N = 20 ES = 2.61 SRM = 2.69
Eshoj H et al 2017 [19] (The Danish electronic version)	Total: N = 62 (1) subacute phase following an anterior shoulder dislocation (2) after Bankart repair 5–7 years ago (3) with self-reported feeling of SI	NA	N = 41 0.97 (14 days) Domains: 0.93-0.96	N = 62 NPRS r = 0.83 OSS r = 0.79	N = 41 MDC = 277.5 Domains: 71.2-161.0 SEM = 100.1 Domains: 25.7-58.1	NA	NA

Study	N	Internal consistency	ICC (interval)	Construct Validity	SEM & SDC/MDC	MCID	SRM & ES
Perrin et al.<br>2017 [20]<br>(The French version)	Total:<br>N = 82<br>21 chronic SI<br>20 surgery<br>41 healthy	N = 41<br>α = 0.95<br>Domains: NA	N = 27<br>0.88 (7 days)<br>Domains: 0.80 to 0.94	N = 41<br>VAS pain r = 0.48<br>N =38<br>Quick DASH r = 0.65<br>Rowe total r = −0.69 N=40<br>Walch-Duplay r = −0.76	N = 41<br>SEM 120.2<br>(5.7%)<br>Domains: 20.0-68.1<br>MDC 333<br>(15.9%)<br>Domains: NA	NA	NA
Basar et al.<br>2017 [21]<br>(The Turkish version)	Total: N = 60 shoulder instability (anterior, posterior or multidirectional)	N = 60<br>α = 0.91<br>Domains: 0.77-0.82	N = 30<br>0.97 (72 h)<br>Domains: 0.83-0.97	N = 60<br>Rowe Score r = −0.57<br>OSIQ r = 0.74<br>DASH r = 0.67<br>WORC r = 0.89	NA	NA	NA
Gottlieb et al.<br>2019 [22]<br>(The Hebrew version)	Total: N = 25<br>arthroscopic repair of SI	N = 25<br>Baseline (0-2 weeks after surgery)<br>α = 0.89<br>Domains: 0.69-0.79<br>Follow-up (7-8 weeks after surgery)<br>α = 0.95<br>Domains: 0.78-0.91	NA	N = 25<br>DASH<br>Baseline r = 0.77<br>Follow-up r = 0.85	NA	NA	N = 25<br>SRM = 1.29
Khaja et al.<br>2020 [23]<br>(The Arabic version)	Total: N = 100<br>Not specified	N = 100<br>Baseline α = 0.85<br>Domains: 0.70-0.79<br>Follow-up (2 months after baseline test)<br>α = 0.91<br>Domains: 0.69-0.87	NA	N = 100<br>DASH<br>Baseline r = 0.79<br>Follow-up r = 0.87	NA	NA	N = 100<br>SRM = 1.45

Study	N	Internal consistency	ICC (interval)	Construct validity	SEM & SDC/MDC	MCID	SRM & ES
Ismail et al.<br>2020 [24]<br>(The Arabic version)	Total: N = 44<br>SI - traumatic or atraumatic (anterior, posterior or multidirectional)	N = 44<br>α = 0.91<br>Domains: 0.56-0.87	N = 44<br>0.96 (within one week)<br>Domains: 0.89-0.98	N = 44<br>DASH r = 0.60<br>ASES r = 0.62	N = 44<br>SEM = 90.2<br>Domains:<br>11-49.4<br>MDC = 250<br>Domains: 30.4-136.9	NA	N = 24<br>SRM = 2.94<br>ES = 3.17<br>Domains: NA
Torres et al.<br>2022 [25]<br>(The European Portuguese version)	Total: N = 81<br>SI (anterior, posterior or multidirectional)	N = 81<br>α = 0.97<br>Domains: 0.77-0.93	N = 50<br>0.98 (72 hours)<br>Domains: 0.97-0.99	N = 81<br>QuickDASH r = -0.79<br>SF-12:<br>PCS r = 0.67<br>MCS r = 0.35	N = 50<br>SEM = 3.10%<br>Domains:<br>3.37-4.52%<br>MDC = 8.60%<br>(individual level)<br>Domains: 9.35-12.53%<br>MDC 1.22%<br>(group level)<br>Domains:<br>1.32-1.77	NA	NA
Bejer et al.<br>2022<br>(The Polish version)	Total: N = 74 operated due to SI - traumatic or atraumatic (anterior, posterior or multidirectional)	N = 74<br>α = 0.94<br>Domains: 0.90-0.95	N = 71<br>0.99 (48–72 hours)<br>Domains: 0.98-0.99	N = 74<br>SF-36:<br>PF r = 0.72<br>RF r = − 0.62<br>BP r = 0.67<br>GH r = 0.51<br>V r = 0.27<br>SF r = 0.43<br>RE r = 0.45<br>MH r = 0.39<br>PCS r = 0.74<br>MCS r = 0.42<br>QuickDASH r = -0.80	N = 71<br>SEM = 1.41%<br>Domains: 1.82-3.10%<br>MDC = 3.90%<br>Domains: 5.05-8.60%	N=51<br>Anchor-Based Method: 126.43<br>Distribution-Based Method: 174.05	N = 51<br>ES=0.44<br>Domains:<br>0.37-0.45<br>SRM=1.26<br>Domains:<br>0.87-1.26

Measurement properties: internal consistency: α Cronbach's alpha; ICC the intraclass correlation coefficient; construct validity: the Pearson's/Spearman's correlation coefficient; SEM the standard error of measurement; SDC the smallest detectable change; MDC the minimal detectable change; MCID the minimal clinically important difference; sensitivity to change: SRM the standardised response mean and ES the effect size.

NA not assessed; SI Shoulder Instability; n number; % per cent

The questionnaires: ASES the American Shoulder and Elbow Surgeons Shoulder Score; DASH the Disabilities of Arm, Shoulder and Hand Scale; QuickDASH the Shortened version of the Disabilities of Arm, Shoulder and Hand Questionnaire; EQ-5D-5L the EuroQol 5 Dimension 5 Levels; NPRS the Numeric Pain Rating Scale; OSIS the Oxford Shoulder Instability Score; OSIQ the Oxford Shoulder Instability Questionnaire; OSS the Oxford Shoulder Score; Rowe Score Rating Sheet of Bankart Repair; SST the Simple Shoulder Test; SF-12 the Short Form-12 Health Survey; SF-36 v. 2.0 the Short Form-36 Health Survey, version 2.0; PF Physical Functioning; SF Social Functioning; GH General Health; V Vitality; MH Mental Health; RE Role Emotional; RF Role Functional; BP Bodily Pain; PCS the Physical Component Summary; MCS the Mental Component Summary; VAS the Visual analog scale; WOSI the Western Ontario Shoulder Instability Index; WORC the Western Ontario Rotator Cuff Index.
